# Introductory Lectures and Addresses on Medical Subjects

**Published:** 1860-03

**Authors:** 


Introductory Lectures and Addresses on Medical Subjects. delivered chiefly before the Medical Classes of the University of Pennsylvania. By George B. Wood, M. D., L. L. D.

We have received and conned the pages of this volume of the Introductory Lectures and Addresses, of Dr. Wood, with much pleasure and profit, and we are sure that a more worthy memorial of the relation that has for so many years subsisted between the author and the numerous classes of young men who have attended the University could not have been devised. They will also find a hearty welcome with the whole profession, of whatever predilection. And great as the delight must be of those who can indulge in the thousand glorious recollections aroused by the graphic history of scenes and persons once familiar to their daily sight; yet a far higher value must be attached to them, from the powerful stimulus such stirring pictures inevitably give to the pursuit of knowledge. No where can the student meet with a source of impulse that will urge him on to future attainments, and no where could he find more effectual inducements to emulate the most exalted efforts of the great intellects and distinguished leaders of the profession espoused, than in the perusal of these Introductories.

We find that the author has presented through this customary Introductory, the consideration of the important subject of Materia Medica, and as such will be the most valuable part of the work; but the two lectures which give the result of the author's professional observations in Great Britain and the Continent of Europe, have a peculiar interest and value. The whole volume, we trust will not only prove instructive and agreeable to practitioners and students throughout the whole country, but will serve as a serviceable book to such as may be willing to visit the scenes from which so many advantages and so much pleasure and satisfaction may be derived.

